# Charting a Moral Life: The Influence of Stigma and Filial Duties on Marital Decisions among Chinese Men who Have Sex with Men

**DOI:** 10.1371/journal.pone.0071778

**Published:** 2013-08-09

**Authors:** Wayne T. Steward, Pierre Miège, Kyung-Hee Choi

**Affiliations:** 1 Center for AIDS Prevention Studies, Department of Medicine, University of California San Francisco, San Francisco, California, United States of America; 2 Institute of Social Development and Public Policy, Beijing Normal University, Beijing, People’s Republic of China; The University of New South Wales, Australia

## Abstract

**Introduction:**

Stigma constitutes a critical challenge to the rising rates of HIV among Chinese men who have sex with men (MSM). It reduces willingness to disclose one’s sexual orientation and can lead to concurrent sexual partnerships. Disclosure decisions are also affected by cultural norms that place pressures on sons to marry. In this manuscript, we characterize how stigma and cultural factors influenced Chinese MSM’s decisions around disclosure and marriage. We seek to show that MSM’s actions were motivated by moral considerations, even when those choices posed HIV transmission risks.

**Methods:**

We conducted qualitative interviews with 30 MSM in Beijing, China. Interviews were audio-recorded, transcribed, and translated into English for analysis. Transcripts were coded using a procedure that allowed for themes to emerge organically.

**Results:**

Participants struggled with feelings of shame and believed that others possessed stigmatizing attitudes about homosexuality. They had experienced relatively little discrimination because they infrequently disclosed their MSM status. In response to marital pressures, participant had to reconcile same-sex attractions with filial expectations. Their choices included: not being involved with women; putting on the appearance of a heterosexual relationship by marrying a lesbian; or fulfilling family expectations by marrying a heterosexual woman. Regardless of the decision, many rooted the justifications for their choices in the considerations they had given to others’ needs.

**Conclusion:**

The growing epidemic among MSM in China requires action from the public health community. As programs are scaled up to serve these men, it is critical to remember that MSM, who often fear social sanction if they were to reveal their sexual orientation, continue to face the same pressures from culturally normative social duties as heterosexual men. Interventions must find ways to help men navigate a balance between their own needs and the responsibilities they feel toward their parents and others.

## Introduction

HIV is rising dramatically among Chinese men who have sex with men (MSM). Recent estimates suggest an incidence rate of 3.5% to 6.7% [1], which is substantially higher than the rates observed in MSM populations elsewhere [2]. Although direct comparisons of disease impact in the Chinese MSM and general populations are limited, one study of individuals seeking services in a sexually transmitted infections (STI) clinic found that gay or bisexual men had nearly 17 times the odds of being HIV-positive than men who were exclusively heterosexual [3]. Because substantial numbers of Chinese MSM report concurrent male and female partners [4], there is concern that the high burden of HIV disease among them could eventually result in increased disease prevalence among heterosexuals [5]–[9].

Stigma constitutes a critical challenge to addressing this emerging epidemic. Experiences of prejudice and discrimination are linked to higher rates of unprotected sex, family pressures to marry and have children, and deferral of testing and treatment [4], [10]. But to fully understand and address the effects of stigma, its diverse manifestations must be considered. At its core, stigma is a socially shared process by which a society discourages specific behaviors and conditions [11]. Such behavioral regulation is accomplished both through actions between people and through thought processes internal to any one individual. *Enacted stigma* refers specifically to interpersonal acts of discrimination against individuals who have or are perceived to have socially scorned conditions, like same-sex attraction [12], [13]. By contrast, *felt normative stigma* consists of intrapersonal perceptions about the prevalence of stigma in the local community [12], [13]. It is a belief that others harbor hostile feelings toward, or a willingness to discriminate against, individuals with a scorned condition. *Internalized stigma* refers to a person’s own prejudicial beliefs about a condition. If that person possesses the stigmatized condition, then internalized stigma is effectively a form of self-stigma [13]–[15].

For stigmatizing conditions that can potentially be hidden from others (what has traditionally been referred to as a “discreditable stigma” [11]), these three manifestations interact in important ways. If looking only at acts of overt discrimination (enacted stigma), stigma can appear to be relatively low [13], [16]. But the absence of discriminatory acts is often due to the intrapersonal, felt normative and internalized, forms of stigmas. Because individuals with a scorned condition believe that others may hold prejudicial beliefs (felt normative stigma) and because they sometimes personally endorse such beliefs (internalized stigma), they go to great lengths to avoid disclosure of their condition [13], [16]. As a result, they infrequently encounter hostility or discrimination. Unfortunately, although such non-disclosure efforts may protect a person from enacted stigma, they also have significant consequences for mental and physical health [10], [13], [17]. The same relationship among stigma manifestations is likely to exist for Chinese MSM. Research has already shown that such men report relatively low levels of enacted stigma [10], [18]. If felt normative and internalized stigmas reduce willingness to disclose same sex attractions, then this absence of discrimination would effectively be a byproduct of the men presenting as heterosexual in most social situations.

Understanding non-disclosure motivations and behaviors among Chinese MSM is likely to be complicated, however, by other cultural constructs and norms common to Eastern societies. One construct of particular relevance is filial piety. Sons are expected to fulfill family obligations to marry, have children, and continue the family name [19]. A second relevant construct is conversational norms. Sex (and, more generally, risk factors for HIV) are sensitive topics not typically considered appropriate to discuss openly with family and friends [20]. Cultural dynamics have already been shown to influence the kinds of stigma concerns of salience to Chinese MSM. Those who are more focused on individual success (e.g., trying to do things better than others) report higher levels of internalized stigma. By contrast, those who are more focused on collective success (e.g., willing to give up personal pursuits to take care of family) report more felt normative stigma [21].

The intersection of stigma and cultural norms may also alter what choices are made in the wake of stigma by placing enhanced attention on one’s obligations to others. Morality has been described as reflecting “prescriptive judgments of justice, rights, and welfare pertaining to how people ought to relate to one another” [22]. Studies have shown that moral assessments can be influenced by individualist considerations (e.g., concerns for fairness and people’s welfare) or collectivist considerations (e.g., respect for authority, in-group loyalty) [23], [24]. Whereas a decision in other settings not to disclose a stigmatized condition might be motivated more exclusively by one’s own welfare (i.e., avoidance of potential discrimination) [13], such choices for MSM in China are likely also to be influenced by their endorsement and understanding of filial duties (a collectivist moral consideration related to authority and in-group loyalty). This would change how men understand and potentially justify their own behaviors.

In this manuscript, we present findings from qualitative interviews with MSM in Beijing. We seek to characterize how stigma and cultural factors influence men’s behaviors and decisions, particularly around disclosure of MSM status and marriage. Although prior survey-based observations have documented associations between stigma and HIV risk behaviors with male and female partners [4], such methods are not able to capture the nuance and complexity of circumstances that ultimately lead to the observed associations. Through the qualitative data we present here, we seek to show that, despite very different choices about marriage and partners, MSM’s actions are motivated by moral considerations, even when those choices may ultimately pose HIV risks for themselves and their partners.

## Methods

Our description of the recruitment, data collection, and analytic procedures follows the recommendations of COREQ, the consolidated criteria for reporting qualitative research [25]. We conducted qualitative interviews between October, 2009, and January, 2010, with 30 MSM who were living in Beijing, China. Men were referred to the study by two recruiters who were familiar with MSM social networks within the city. They sought out potential participants through organizations serving MSM or in venues where MSM were known to congregate. At any given location, the recruiter would work with a known contact to help identify men to be approached, and would then briefly describe the study and its procedures to those individuals. The need to use recruiters and their contacts is reflective of the current social context for MSM. Men are unlikely to agree to participate if approached directly by an unknown researcher, nor are they likely to respond to flyers advertising a study. Working through recruiters and their contacts allows the initial offer to come from a person that MSM know and trust.

For men interested in participating, the recruiter arranged a time for them to meet individually with a study investigator in a private office at a local university. We sought to obtain a sample that was diverse in terms of age; self-identification as gay, bisexual, or heterosexual; and migration status. These factors were used because prior MSM samples have had an overrepresentation of young men, previous studies have shown that there are substantial numbers of MSM who have sex with both men and women, and earlier research suggests that large proportions of urban-dwelling MSM are individuals who have migrated from elsewhere in the country [4], [26], [27]. To be eligible to participate, an individual had to be 18 years of age or older, be male, and report having had sex with other men. (The use of recruiters and their contacts precluded the calculation of an overall refusal rate, as investigators were not present for the initial contact with prospective participants. But of the men who presented to the investigative team for interviewing, only one was not enrolled in the study. In that case, the man was believed to be gay by other MSM, including the recruiters and their contacts, because he worked for an organization that offered services to MSM. However, he revealed to an investigator that he was in fact exclusively heterosexual and, thus, not eligible for inclusion in the study.) To preserve anonymity, participants were asked to provide verbal consent, which was documented in study records by the investigator conducting the interview. The men received 150 Yuan (equivalent of approximately US$24) to reimburse them for their time. All procedures for the study, including the use of verbal consent procedures, were approved by the institutional review boards (IRB) at the University of California San Francisco and Beijing Normal University.

### Interview Procedures

Each participant was interviewed once for approximately one hour. Interviews were audio-recorded, subsequently transcribed and, for interviews in Mandarin Chinese, translated into English for analysis. Transcription and translation were conducted by study staff and then reviewed by a study investigator (PM) to ensure accuracy.

Interviews covered a variety of topics related to stigma. They asked men to talk about experiences of discrimination (enacted stigma); perceptions of attitudes about homosexuality among family, friends, and colleagues (felt normative stigma); men’s personal feelings about their sexuality (internalized stigma); their approaches to managing stigma; decisions about disclosing to family, friends, and colleagues; and reactions to any disclosures that had occurred. To help build rapport during the interview and to provide context for understanding a participant’s history with stigma, the investigator opened each interview by asking the participant to talk more generally about his life and to describe how he first came to discover that he was interested sexually in other men and to label his identity and/or behavior. The interview guide specified the broad topics to be covered and offered suggestions for probes and follow-up inquiries. However, the investigator conducting the interview was free to adapt the questions to facilitate a more normal conversational flow, to follow-up on stigma-related details reported by a participant, or to skip sets of questions that were no longer applicable based on the information that a participant had reported earlier.

To ensure that the interview guide elicited desired information, the first three interviews were conducted using both Mandarin Chinese and English. Participants for these interviews consented to have two male interviewers (WS, PM) in the room. One of the interviewers (WS) holds a PhD in psychology, is a faculty member at a university in the United States, and is experienced in both qualitative and quantitative research on stigma. The other interviewer (PM) holds a PhD in sociology, is a faculty member at a Chinese university, speaks and writes Mandarin Chinese, and had conducted previous research on MSM communities in China. Their credentials were explained to participants prior to the interview. The conversation proceeded in English, but switched to Chinese if needed for clarity or to enable a participant to fully answer a question. These initial interviews allowed the investigators to determine if the concepts being assessed made sense to participants and if the kinds of narratives being elicited were consistent with the objectives of the study. After determining that the interview guide worked as intended, the remaining interviews were conducted exclusively in Mandarin Chinese by the investigator fluent in that language (PM). While data collection was ongoing, all three investigators (WS, PM, KC) met regularly to discuss information being learned in the interviews, to identify any emerging themes, and to decide if emerging themes warranted greater follow-up in subsequent interviews. Data collection was stopped after 30 total interviews because the investigators determined that data saturation had been reached.

### Analysis

Interview transcripts were coded using a procedure developed by Corbin and Strauss that allows for themes to emerge organically based on the content of the interviews [28]. Initially, a subset of interviews (n = 2) was analyzed collectively by the three investigators (WS, PM, KC) to allow the team to identify a preliminary set of codes and to agree upon the kind of text that appropriately fit under each of the codes. From this work, a codebook was drafted. Investigators then independently read and coded the remainder of the transcripts. At least two investigators reviewed each transcript. Disagreements in codes were resolved by discussion. Throughout this process, the investigators met weekly to review the transcripts that had been coded and verified, to suggest new codes, and to ensure ongoing consistency in how the codes were understood and applied. We were not able to share the codes and emerging themes with participants because men had been enrolled anonymously and, per the requirements of our approved IRB protocols, their contact information was not retained.

The coded interview transcripts are securely housed on servers at the UCSF Center for AIDS Prevention Studies. Inquiries about accessing the data can be made by contacting the first or senior author of this paper. For the protection of the participants, the interview transcripts are not available in a publicly accessible archive. Although identifiers were redacted from the transcripts, the detailed narratives potentially make it possible for some participants to be recognized if a reader happened to be familiar with the specific events described in the interviews.

## Results

Participants varied in their demographic characteristics. Most were between the ages of 20 and 39 (mean: 33.3; range: 21–52). One-fifth of the men were or had been married (16.7% currently married, 3.3% divorced). A little over half (53.3%) had completed college and the vast majority (90%) was employed. Most participants (73.3%) had migrated to Beijing. All reported being Han, which is the majority ethnic group in China.

### Manifestations of Stigma

Of the various manifestations of stigma, the two intrapersonal forms were highly salient. Many participants had struggled with feelings of shame about homosexuality (internalized stigma), particularly around the time that they first realized they had attractions to men.

Participant: *I was one of those early maturing, quite early maturing, children. And when I was 14, I did that kind of thing [had sex] with a fellow student, a male student, at [participant names an educational facility]. […]*

Interviewer: How did you feel?

Participant: *All kinds of feelings–there was this nervousness, then surprise, then happiness. That’s really how it was. […] At the time, I was really at a loss, really at a loss. I couldn’t understand why I would want that kind of thing. Then there was the matter of whether this behavior was right or wrong.* –Participant 22 (38 years old, college education, originally from Beijing, currently unmarried)

*He [a college classmate with whom the participant was friends when he was 20 years old] told me about homosexuality, plus he told me he liked me. I felt–in any case, how do I put it?–I felt it was a very strange situation. I said, “Why do you like me?” I felt it was a situation that was very, very hard to believe. And then, at the time, I rejected him. I couldn’t accept it. […] At the time, I felt all gay men had AIDS. I felt that they were all people infected with AIDS. I said, “I didn’t want to be around them.” It was that kind of mentality.* –Participant 25 (37 years old, postgraduate education, originally from Beijing, currently unmarried)

This same participant subsequently continued to reflect on the same period in his life:

*I definitely had this conflict because I definitely liked it. I definitely liked homosexual interaction or even physical contact. I was completely willing. If I liked a person, I would definitely be willing to have this kind of interaction. But I was also scared. At the time, I was very childish. […] I just thought that this kind of person [MSM] was equivalent to AIDS. This kind of person, I thought that, if he had AIDS, I definitely wouldn’t be willing to have anything to do with him. So I withdrew and rejected it. I would hide from them [other MSM] and walk away.* –Participant 25

Participants also held perceptions that others in the community possessed stigmatizing attitudes about homosexuality (felt normative stigma).

*Some people are more open-minded and say that it [being gay] is not a big deal when they find out. But other people will turn on you and start asking what kind of person you are. They don’t think well of you. That’s how I think it is.* –Participant 13 (27 years old, senior high school education, migrated to Beijing, currently unmarried)

Interviewer*: In general, how do you think society perceives gay people? What kind of attitude do they have?*

Participant*: They think these people are sick.*

Interviewer*: Do you think most individuals feel that way?*

Participant*: Yes, or most will use the term ‘abnormal’ [to describe gay people].* –Participant 5 (27 years old, college education, migrated to Beijing, currently unmarried)

By contrast, there were fewer reported acts of discrimination or prejudice (enacted stigma). One example came in an interview with an older participant, describing an event that had occurred in the early 1990s when he met a sexual partner at a public sex venue:

*So I met this guy and we both thought, “Ok, let’s do something.” So we crossed to the other side, by the river, and there we started to hug, and he started to unfasten my belt. It was in winter, and I suddenly saw someone in front of us with an electric torch. I was shocked. I just pushed him [the sexual partner] back. At that moment, there were two guys standing in front of us. They immediately separated us and started to ask questions: “Who are you? Do you know this guy?” I forgot what I said but because he [the sexual partner] had unfastened my belt, I needed to hold it. I also had an overcoat that I was holding. And the two men thought I might have a weapon on me. So they hit my arm and it caused me to let go of my trousers and my trousers start to fall. When that happened, he started to beat me while saying, “Ah! You are ‘rabbitting’” [an expression to describe homosexual behavior]. He started to use his belt to hit me. And I had my overcoat, so I used that overcoat to protect my head. This lasted about two minutes and then… I was just in shock and saying, “Did I do something against the law?” or something like that. They asked me to be quiet and started to take notes. “Who you are? What is your ID number? Do you have a job? Where do you live?”* –Participant 3 (48 years old, college education, originally from Beijing, currently unmarried)

But this example was relatively unusual. In many of the interviews, there were no stories of overt discrimination.

### The Interaction of Stigma Manifestations: Felt Normative Stigma Led to Little Disclosure and Limited Exposure to Enacted Stigma

The pattern of findings appeared to be due to men’s limited disclosure of their same-sex behavior. Participants believed that family, friends, and colleagues would not be supportive of homosexuality (felt normative stigma) and, thus, chose not to tell these individuals about their romantic attractions. When explaining decisions not to disclose, men spoke of how they anticipated enactments of stigma if others knew that they (the participants) were MSM:

Interviewer: *Have you ever experienced discrimination?*

Participant: *If I revealed my identity, I would definitely experience discrimination from a lot of people. A few days ago I saw some statistics in the newspaper about AIDS infections. In China, some 30% of AIDS transmissions are through blood donations, and 30% is because of sex between men. The surveys show that in society, the 30% in the blood group will get compassion from society and the other 30% that got it through sex between men will be discriminated against. It’s the same. If I were to reveal it, I would definitely receive discrimination. So I have to disguise myself. I don’t want to let myself be discriminated against so I’ll keep hiding it in the long-term.* –Participant 17 (26 years old, senior high school education, migrated to Beijing, currently unmarried)

Interviewer: *Where would discrimination occur?*

Participant: *Well, for example…family members. Of course I think that in the end, family is tolerant. But despite that, it [knowing a person is MSM] would change how they perceive you. Let’s say there were some coworkers or neighbors that I wasn’t especially close with. If they found out my identity, I think they would look at me as if I were a freak.* –Participant 21 (37 years old, college education, originally from Beijing, currently unmarried)

Interviewer: *Why haven’t you told anyone?*

Participant: *I think there’s still some pressure. Even though the environment and the people wouldn’t be too averse and would accept me calmly, honestly speaking, you’re still worried. Will friends distance themselves from you? Will they look at you differently? Mostly, you’ll still be a little worried. This worry makes you decide that it’s best not to say anything.* –Participant 4 (34 years old, college education, migrated to Beijing, currently unmarried)

### The Role of Culture: Expectations of Marriage

Reflecting cultural norms about the duties of sons, participants also spoke frequently about the pressures of marriage:

*Everyone faces the marriage problem. To be on the verge of entering the stage of marriage is very important. It’s also a brutal problem. Say I want to have this life [live openly as a gay man]. It’s like talking about the Milky Way. It doesn’t exist in China. You’re forced into marriage. This is one way in which the contemporary life of China’s MSM is sad.* –Participant 4 (34 years old, college education, migrated to Beijing, currently unmarried)

*Because my parents are getting older, every parent hopes their own child will have a family and a career, this is a tradition in China, and then parents want children to live a life of happiness. I feel that this is unquestionable. If I don’t want to get married, my parents will keep worrying about me nonstop. They will incessantly think about it. Beijing is considered this small area, these elderly fellows and elderly ladies. Retired elderly fellows and elderly ladies frequently talk with each other,* [participant imitates exchange between elderly men and women] *“Has your son gotten married yet? Has your daughter gotten married yet? Who is the fiancé? Etc.” They will ask these kinds of questions, and once they ask my father, my dad says, “My son has not gotten married yet.” And people just think, “So old and still not married.” It must cause my father to lose face in front of his old companions. He will feel that his son is useless, feel like his son can’t even get a wife.* –Participant 25 (37 years old, postgraduate education, originally from Beijing, currently unmarried)

The amount of pressure men experienced was thought to differ over time. Some younger participants felt pressures might grow stronger as they aged.

Interviewer: *If you get married, would it be because of your parents? Or someone else? Why might you choose marriage?*

Participant: *Yes, probably for a family reason. My parents will definitely want an explanation. I think in the next few years it’s possible. It’s hard to say.*

Interviewer: *Do you think there’s any way to avoid marriage?*

Participant: *I might be able to, but I can only keep on delaying it like this–that is, when I go home, I rarely go home. The minimum is once a year for one day during the New Year and then I quickly return to Beijing.*

Interviewer: *Do they [your parents] call you to press you about when you’re getting married?*

Participant: *They do when they call. But I only say that I’m busy at work and have no time. I have to keep delaying it and delaying it.* –Participant 17 (26 years old, senior high school education, migrated to Beijing, currently unmarried)

Other, older men found that pressures subsided after a certain age.

*After I turned 35, the feeling my family gave me was, “You’re not young anymore. We won’t push you anymore. If you don’t want to [get married], forget it. You can be single. Of course, if it’s in your fate, we still hope that you can get married. If it’s not in your fate, then you’ll just be single.”* –Participant 21 (37 years old, college education, originally from Beijing, currently unmarried)

### The Intersection of Stigma, Marriage Expectations, and Disclosure Decisions

In response to the pressures from family and friends, men felt compelled to make decisions about how to reconcile their same-sex attractions with societal expectations. Their choices included: not being involved with women, either by living life as an openly gay man or by finding ways to avoid being linked romantically to women; putting on the appearance of a heterosexual relationship by marrying a lesbian; and marrying a heterosexual woman. Regardless of the decision, many justified their choices as being the correct pathway for them based on considerations of their own and/or others’ needs. In the sections that follow, we describe these different choices and what motivated men to pursue them.

#### Decision not to be with a woman

Half of the participants (n = 15) said that they did not plan to get married. Many of these men felt it was unjust to marry women. In the interviews, they framed their thinking in terms of their own welfare and the welfare of others, including a hypothetical female sexual partner.

*I don’t plan on getting married. Getting married won’t just hurt me, it will hurt other people too. That is how I feel. If I were to marry a girl, could I give her a happy life or anything like that? I am not willing to get married when I think about this.* –Participant 13 (27 years old, senior high school education, migrated to Beijing, currently unmarried)

*I said very firmly, I said that I might be gay so I won’t marry. I said, “This is my life, I feel like this is very good. I do not choose marriage.”* –Participant 11 (38 years old, college education, originally from Beijing, currently unmarried)

*I think marriage is meaningless for me. The other reason [not to marry] is I don’t want to make a girl–because she is innocent, not guilty. If I were to get married, maybe it would hurt her.* –Participant 2 (31 years old, post-graduate education, migrated to Beijing, currently unmarried)

*My viewpoint is very extreme in terms of marriage between MSM and heterosexuals. I resolutely condemn it, do you understand? […] There are people who say it’s in order to protect oneself because of the so-called environment, so they have to get married to a woman. This I resolutely condemn, I object to these kinds of things. I feel that it’s not ethical. […] To sacrifice a woman in exchange for oneself, to protect oneself, I feel like this is an extremely unethical thing.* –Participant 9 (52 years old, college education, originally from Beijing, currently unmarried)

Because these men were not necessarily willing to disclose their sexual orientation, they sometimes had to devise strategies to deflect efforts from family or friends to introduce them to potential female partners, as reflected in this quote from one participant:

*[When introduced to women,] I first say, “I’m so grateful for the introduction. Things require that we take it one step at a time so let’s just be friends first.” But I don’t get in touch with them frequently, about once or twice a month. Then they just think we are getting along like normal friends.* –Participant 4 (34 years old, college education, migrated to Beijing, currently unmarried)

Another man noted that considerations of others might eventually lead him to disclose his MSM status, at least to close friends:

Participant: *Having to deceive your own friends and keep from them your thoughts and feelings is harder. I think it’s okay with colleagues because you don’t have as much affection for them. If you need to lie, then lie. There isn’t much stress in it. But lying to good friends is much harder.*

Interviewer: *So do you hope that one day you can tell them?*

Participant: *I might be able to. If you regularly talk to some of your friends, you’ll have to tell them because they’re always worrying about you. They worry about why you don’t have a girlfriend, why you don’t…*

Interviewer: *Get married?*

Participant: *Right. If you don’t want them to worry, then you have to tell them. Otherwise they’ll always be nagging you.* –Participant 20 (34 years old, college education, migrated to Beijing, currently unmarried)

However, as reflected in the experiences of a third participant, disclosure did not necessarily bring an end to pressures and the need to constantly defend a decision not to marry:

Interviewer: *So your mother* [to whom the participant had disclosed his sexuality] *still wants you to marry a girl?*

Participant: *Yeah, she still continuously asks me to marry a girl. Even though my mother understands that I may never marry a girl, as a mother, her understanding is just up to this level, up to, “As long as I keep talking, keep going on about it, maybe you will understand.” She also knows that I would consider her feelings, I would, I would compromise to some extent, she probably just doesn’t think about whether I’d really be happy [if I got married to a woman][…] She thought two boys together wouldn’t gain any kind of happiness, probably you could be happy temporarily, but she thinks homosexuality might just be a temporary thing, and everybody needs to get married. I’d say her understanding [of homosexuality] is just up to this level.*

Interviewer: *Do you think this puts a lot of psychological pressure on you?*

Participant: *A little bit–probably I didn’t think about this before. Later, I gave it a lot of thought, and I may have to consider my mother’s feelings. Thinking about it, my mother is already 70 years old this year. I think if I keep doing this with her, it’s like pouring cold water on her, making her dispirited. I can’t really bear doing that to her, but I still won’t get married. This is the fact that I can’t change.* –Participant 29 (39 years old, college education, migrated to Beijing, currently unmarried)

#### Decision to marry a lesbian

A second pathway involved marrying a lesbian woman, a strategy that would allow men to avoid disclosure of their MSM status to family and friends but not require they deceive the woman who would become their legal wife. This choice was observed less frequently in our sample, with active efforts to engage in the strategy observed among two relatively older (age 35+) participants. One individual stated that he currently had a lesbian girlfriend, in addition to a boyfriend, and was actively seeking a lesbian wife. His choice was framed in terms of considerations to his parents, specifically, to enact a married life in order to honor parents’ wishes.

*I feel that at this age, it is the time with the greatest pressure. I feel that if I can bear through [age] 40, then it won’t be as intense, but right now it’s the greatest. My family urgently wants me to marry this year, so I keep looking for a lesbian, and then I hope I can find one, and then get married. […] I feel my parents are too old, about 70 years old. I don’t want them to pass away before seeing me get married. I hope that before they pass away, they can see me get married, see me have a prosperous life. I feel that they will be at peace, they can also be very, very happy when leaving. I feel that if this happens, it’s an explanation: at least don’t let your parents worry.* –Participant 25 (37 years old, postgraduate education, originally from Beijing, currently unmarried)

This same participant described his approaches for meeting lesbian partners:

*I looked on the Internet, through Internet channels. In the past, if I felt that the lesbians I found were not suitable, they would help introduce me to their friends.* –Participant 25

He also noted that the search for a lesbian wife was something he coordinated with his current boyfriend.

*When I am looking for a lesbian, I am simultaneously looking for him. If there is someone suitable, we will just go and meet them together. This evening, we are going to meet a pair of lesbians.* –Participant 25

A second participant reported having successfully presented himself as married to a lesbian.

Participant: *I had a marriage in form but it wasn’t lawful. You could go online by that time. It must have been around [the year] 2000. You could go online to search. At the time, my family couldn’t give me real pressure because I was only home seven days a year. I was still scared of them and felt really guilty. I felt like I had to apologize to my parents because they just wanted me to get married. I thought I should help them stop worrying and be a little happier, so I went online and found this woman.*

Interviewer: *Was she a lesbian?*

Participant: *Yes, a lesbian.*

Interviewer: *But it wasn’t successful?*

Participant: *It was.*

Interviewer: *Did you two get married?*

Participant: *We didn’t get a marriage certificate but we told our families that we did. We’re not lawfully married but they think we are.* –Participant 26 (41 years old, college education, migrated to Beijing, currently characterizes himself as married but does not technically have a marriage certificate)

He went on to note that, although well intentioned, the strategy didn’t necessarily lead to an end to family pressures.

Participant: *I haven’t solved any big problems. When I got married, the first two to three years were great. I went home with her every year and also visited her family. But after one or two years, my parents wanted me to have a kid.*

Interviewer: *So the pressure is back?*

Participant: *The pressure is even bigger. Getting married, it’s so simple, it should actually make me happy. If my parents said, “We’re satisfied,” then I’d be satisfied too. I had no idea that they wouldn’t be satisfied at all. That’s because the reason they hoped for me to get married was to have a child. That’s more important to them. But I can’t do that because this lesbian and I have already agreed to not have children. I can’t force her to have a baby and she won’t agree. That’s why it’s even more troublesome now.* –Participant 26

#### Decision to marry a heterosexual woman

A third pathway chosen by men was to marry (or plan to marry) a heterosexual woman. Four participants indicated that they were already married to heterosexual women, one stated that he was divorced, six indicated that they planned to get married in the future, and two said they were currently still undecided about whether to marry. As with the other choices, men justified this decision in terms of perceived responsibilities. However, their explanations often reflected continuing tension, with them weighing the duty they felt toward parents against other considerations. The following examples each come from participants who expressed no personal enthusiasm for marriage, but who felt their responsibilities to their parents had to take priority.

Interviewer: *And what do you think would happen if you told your parents [that you are gay]?*

Participant: *I can’t say but it would be a very, very serious thing. Very, very.*

Interviewer: *What might happen to you?*

Participant: *To me? I don’t worry myself. I worry about them.*

Interviewer: *What do you think would happen to them?*

Participant: *I don’t think they would kill themselves, but they would be heavily heartbroken.* –Participant 1 (29 years old, college education, migrated to Beijing, currently unmarried)

This same participant then went on to say:

*This [marriage] might be their [my parents] last expectation of me. […] I am really tired of pretending. Maybe I only need to pretend with a straight girl that I am straight guy, and cut off my relations with all my gay friends. I will try, I think.* –Participant 1

Two other participants expressed similar sentiments:

*It’s impossible [not to get married]. It completely comes from my responsibility. I’m going to get married out of responsibility to my parents. As for my true feelings about it, I don’t really yearn to get married or long to start a family with a woman. I don’t have those feelings. But what I think more about is satisfying my parents’ wishes. It’s a bit of filial piety towards them. After all, my parents are old and have a lot of desires regarding this. Requiring that I get married satisfies them.* –Participant 7 (29 years old, junior high school education, migrated to Beijing, currently unmarried)

Interviewer: *Why did you recently feel that you had no choice but to get married?*

Participant: *Because I was getting older. But I think the main reason why I got married was not for myself, but for my parents.*

Interviewer: *How do you think it would be if you weren’t married?*

Participant: *They wouldn’t be angry, but they would perhaps feel that there was some part of a task that they failed to complete in life. If after marrying, I [had] this kind of life, a family-oriented life, maybe they would feel that this period of my life was worth something. The remaining part I would continue myself, whether it be producing offspring or fulfilling their hopes and dreams on my behalf and so on.* –Participant 14 (30 years old, college education, originally from Beijing, currently married)

Men who were–or intended on being–married acknowledged that this choice was complicated and that it left them at risk of concurrent partnerships with men.

Participant: *At this point, my sexual orientation heavily relies on men when it comes to sexual desires, right? So even after I’m married to a woman, I might still have a need for men.*

Interviewer: *You said that this would be very painful.*

Participant: *Yes, of course that’s a painful process. If you marry a woman, you’ll have a lot of family responsibilities. Also, think about it. You still have needs and demands when it comes to men. It’s a very conflicting issue. You have both feelings and pressures. You have to gradually deal with it. What do you do? There’s nothing to be done. As we say in Chinese, there’s nothing to be done.* –Participant 7 (29 years old, junior high school education, migrated to Beijing, currently unmarried)

*I am trying to change. But it’s definitely not what I anticipated. I felt confident that, if the marriage were real, perhaps I would change this kind of life [no longer be MSM]. But after living this kind of life, I found out that this change was not easy. Furthermore, I read a lot of books, some that talked about how certain people are innately gay. Perhaps I am one of those people. I don’t have any feelings for my wife. Perhaps I am with her solely for the sake of responsibility. […] Whenever I come home, not only do I have to play a role, but I also have to tell my parents how happy we are together.* –Participant 14 (30 years old, college education, originally from Beijing, currently married)

This same participant continued later:

Participant: *I think it would be best [to have a boyfriend]. But if I were to get divorced again, from my point of view, the pain would be great.*

Interviewer: *What if at some given time in the future, you meet someone that you really like through your gay friends? What choice would you make?*

Participant: *Maybe I would get a divorce, but I don’t think that I would. Perhaps we could have a secret relationship. There are a lot of stories like this where perhaps the wife has one lover, but you, yourself, have many.* –Participant 14

## Discussion

The findings from our qualitative interviews with Chinese MSM are consistent with the pattern of stigma manifestations found in other studies [13], [16]. Men reported relatively few experiences of discrimination. But this was due largely to their unwillingness to disclose their sexual orientation, a decision that was motivated by the perception that others would judge, and potentially discriminate against, them for having attractions to men. However, the interviews also highlighted the strong role that cultural constructs, particularly filial piety, played in men’s choices [19]. For many, the decision not to live openly as a gay man was not simply about the avoidance of discrimination, but also something born out of their considerations to family members or other individuals.

The impact of these cultural constructs was evidenced in the ways in which participants justified their decisions. Despite very different choices, men repeatedly turned to explanations that grounded their actions in social responsibilities and moral considerations [23], [24]. For men who did not want to have a wife, emphasis was placed not only on responsibility to themselves but also on the harm that they felt they would do to a female partner (i.e., individualist moral consideration for the woman’s welfare). By contrast, men who chose to marry women placed greater emphasis on the need to honor their parents, often struggling with the fear that not marrying would bring shame or disappointment to their mothers and fathers (i.e. collectivist moral considerations for respect for authority and in-group loyalty). A small subset of these men attempted to strike a compromise by seeking a lesbian as a wife. For the limited number of men who had chosen this strategy, success was mixed. It provided an avenue for fulfilling parental expectations to marry without posing perceived harms to the female partner. But it did not eliminate all family pressures, such as expectations to have children. Other participants who had (or planned to) marry heterosexual women were left at a loss for how to be faithful to their wives, recognizing that they might ultimately need male partners but not seeing a way to avoid the lifelong tension between family obligations and personal desires.

Taken together, these findings complement the kinds of associations observed in quantitative studies of stigma. Prior research has shown that internalized stigma concerns are salient for Chinese MSM with a more individualist orientation whereas felt normative stigma concerns are salient for those with a more collectivist orientation [21]. Our findings suggest that individualist and collectivist moral considerations also shape the choices MSM make in response to stigma perceptions and culturally prescribed duties, particularly when making decisions about marriage.

It is of course by no means unique to Chinese culture to hear of MSM feeling pressure to marry women. But whereas modern accounts in other societies may situate these experiences more overtly in stigma [29], [30], men’s reasoning in our interviews was notable for the repeated attributions to broader social duties. This is not to say that disapproval of homosexuality played no role in men’s lives. The fact that a society views a man’s duty as including marriage to a woman is itself reflective of the differing value placed on different sex and same sex relationships. But for the participants in our study, their thinking about marriage was often not characterized as a response to stigma, even when they acknowledged perceptions of stigma elsewhere in their interviews. Instead, men understood themselves to have broad social obligations. And society’s lack of acceptance of their homosexuality left them with an unusual challenge of having to reconcile their sexuality with those duties.

These findings have important implications for addressing the emerging HIV epidemic among MSM in China. First and foremost, they caution against blaming MSM for the complicated situations in which they may find themselves. A variety of studies have noted that rising rates of HIV among MSM could pose a risk to wives and girlfriends–and, by extension, to the broader heterosexual population [5]–[9]. But, as reflected in the findings presented here, the concurrence of male and female partnerships is not necessarily a result of neglect or lack of concern. Rather, Chinese MSM struggle with how best to honor their duties. Their selection of a female partner, despite the absence of attraction, often comes from sincere respect for their parents. In turn, this suggests that stigma reduction and HIV prevention interventions cannot focus simply on constructs like self-acceptance. A man can be at peace with his homosexuality but still feel pressure to marry as a part of social duties. Therefore, interventions will likely need to incorporate considerations of family and help men to develop strategies for negotiating filial obligations. This is not necessarily an easy task given the importance of family and the pervasive influence of cultural norms. But the fact that substantial numbers of men in our sample were choosing not to marry while maintaining contact with family suggests that success is possible. In light of this observation, one potential direction would be to use peer-based interventions in which men who have successfully found ways to mitigate pressures to marry could model their approaches for men who are less sure of how to handle such challenges.

Our findings are limited by several aspects of the study design. First, because we recruited only in Beijing, it is possible that the results do not generalize to MSM in other parts of the country. We note, however, that Beijing’s cosmopolitan status and availability of jobs makes it an attractive destination for migrants. Thus, the men in our sample hailed from diverse regions in the country. Second, we worked through recruiters and their contacts to identify participants. This methodological approach was driven by some of the very realities we sought to study. The potential for discrimination and prejudice makes men less likely to agree to participate in a study if approached by an unknown researcher. The use of recruiters enabled us to reach men who might otherwise have refused to participate. But the recruitment approach also has limitations because it relies on specific recruiters’ networks of contacts, which may or may not reach to all subgroups of MSM. To mitigate this risk, we used recruiters of different ages with distinctly different contact networks. Nevertheless, we cannot fully guarantee that our methods reached all types of MSM. Third, as a qualitative study, we sought to gather enriched, contextual data from a small sample of men. Such research allows us to say something about the range of experience, but precludes us from drawing inferences about population-wide trends (e.g., relative prevalence of the different types of experiences). Future work will need to use other methods, such as quantitative surveys, to assess the prevalence of different manifestations of stigma and to test for the significance of associations among stigma manifestations, cultural factors, and HIV risk behaviors.

The growing epidemic among MSM in China requires action from the public health community. As programs and interventions are scaled up to serve these men, it is critical to remember that MSM face the same pressures from culturally normative social duties as heterosexual men. Interventions must find ways to help men navigate a balance between their own needs and the responsibilities they feel toward their parents and others.
